# Mothers' Perception and Healthcare Seeking Behavior of Pneumonia Children in Rural Bangladesh

**DOI:** 10.1155/2014/690315

**Published:** 2014-02-23

**Authors:** Farzana Ferdous, Fahmida Dil Farzana, Shahnawaz Ahmed, Sumon Kumar Das, Mohammad Abdul Malek, Jui Das, Abu Syed Golam Faruque, Mohammod Jobayer Chisti

**Affiliations:** ^1^Centre for Nutrition and Food Security (CNFS), International Centre for Diarrhoeal Disease Research, Bangladesh (icddr,b), 68 Shaheed Tajuddin Ahmed Sarani, Mohakhali, Dhaka 1212, Bangladesh; ^2^Department of Clinical Trial and Clinical Epidemiology, Graduate School of Comprehensive Human Sciences, University of Tsukuba, Japan; ^3^School of Population Health, The University of Queensland, Brisbane, Australia

## Abstract

We describe mothers' perception about signs and symptoms, causes of the illness, and healthcare seeking behaviors related to pneumonia and express the major modifiable barriers to seeking timely treatment when their under-5 children had pneumonia in rural Bangladesh. Using focus group discussion, we understood mothers' perception and healthcare seeking behavior of childhood pneumonia. Although mothers described pneumonia as a serious life threatening disease in young children but most of the mothers (*n* = 24) could not diagnose whether their child had pneumonia or not. Environmental factors such as dust particles, spread from coughing mother, and drinking cold water or playing with water were perceived as the causes for pneumonia. Three common barriers noted were as follows: illness was not perceived as serious enough or distance from healthcare facility or lack of money at household for seeking treatment outside. Most of the rural mothers did not have knowledge about severity of childhood pneumonia.

## 1. Introduction

Acute lower respiratory infections (ALRI), particularly pneumonia, are the leading and largest single cause of mortality among <5-year-old children in developing countries [1]. The problem is responsible for 18% of the annual 7.6 million deaths in this age group [1]. Moreover, 95% of all these pneumonic deaths occur in the developing countries [2, 3]. Among the under-5 children who die in Bangladesh each year, 14% are due to pneumonia [2]. There are an estimated 150 million new cases of childhood pneumonia reported each year globally, including 61 million cases in Southeast Asia [4]. Of these, 11 to 20 million are severe enough to be life threatening and to necessitate hospitalization in developing countries [4].

World Health Organization (WHO) has classified pneumonia as severe or very severe based on clinical presentation [5]. Standard management of severe pneumonia requires hospitalization for supportive treatment including oxygen therapy, airway suctioning, fluid and nutritional management, antimicrobials, and careful monitoring [5]. Appropriate health care for pneumonia in rural community is very critical due to inadequate number of healthcare facility. Moreover, lack of knowledge about the danger signs and symptoms of pneumonia among the primary caregivers is another cause of delayed seeking care for childhood pneumonia, which could even be life threatening [6, 7]. Several studies have examined the prevalence, health consequence, and clinical management of pneumonia however, inadequate number of literatures has reported the perception, especially of rural community mothers about pneumonia and its signs and symptoms [8–10]. A recent hospital based published data revealed that only 7% caregivers of pneumonic children had a good understanding and 51% had poor understanding about the clinical signs of pneumonia [11]. However, community based information is needed regarding mothers' ideas about causes of childhood pneumonia, and the healthcare seeking behaviors of mothers for their child's pneumonia. Additionally, identification of barriers that impede them from taking appropriate care is critically important. Thus, the present study aimed to understand mothers' perception about signs and symptoms, causes, and healthcare seeking behaviors related to pneumonia when their children had pneumonia. Additionally, the study aimed to identify major modifiable barriers in seeking timely treatment of pneumonia for under-5 children in rural Bangladesh.

## 2. Materials and Methods

### 2.1. Study Design

This study followed an exploratory design.

### 2.2. Study Site

The study centered in a subdistrict of rural Bangladesh named Mirzapur, under Tangail district where people are living with poverty and low school attainment. Mirzapur subdistrict in Tangail district is located about 60 km north of Dhaka, the capital of Bangladesh spanning an area of 374 sq km. The subdistrict consists of 1 urban municipality, 14 unions (lowest administrative unit in rural Bangladesh), and 219 villages. Total population is nearly 400,000; half of them are male. Population density is 981 per sq·km. In Mirzapur, there are 80,000 households; average family size is 4.5 persons. Mirzapur has an average literacy rate of 33% (7 years of schooling). Source of drinking water of 90% dwelling households is tube well. Thirty-six percent of the dwelling households have sanitary toilets; however, 4% have no toilet facility. Among the dwelling households, over 60% have electricity while vast majority of the population are cell phone users. Agriculture is the main occupation for men, while women remain busy mostly with household activities. Rice and jute are the main crops; while other seasonal crops are vegetables including green leafy ones, potato, and mustard seeds. Mothers mostly walk to reach the health facilities. Other transports are cycle rickshaw, motor bike, cycle trailers, improved cycle trailers, country boat, and bus. A field site of International Centre for Diarrhoeal Disease Research, Bangladesh (icddr,b) is located there with a demographic surveillance system (DSS); icddr,b has its collaborative research infrastructures there for more than 25 years.

### 2.3. Kumudini Women's Medical College and Hospital

The study participants were the mothers of pneumonic children who were hospitalized in a large tertiary level facility having 750 beds. It is a charitable institution providing low-cost health services to the surrounding rural population. The hospital has separate outpatient and inpatient departments for pediatric patient population. On an average, over 100 children of pediatric age report to the outpatient a day while 10–15 children are hospitalized in the inpatient with various pediatric problems including pneumonia, severe pneumonia, very severe pneumonia, or severe diseases. All hospitalized children are assessed by the attending physicians, who take medical history, perform clinical examination, and determine management plan. Children diagnosed as cases of pneumonia receive empiric broad spectrum antimicrobial therapy which usually has coverage against likely bacterial organisms causing pneumonia. Those who have bronchospasm receive bronchodilators inhalation (administered by means of nebulizer metered-dose inhaler) after bronchodilator responsiveness test; however, those with increased work of breathing receive supplemental oxygen with a nasal cannula. Naso-pharyngeal suction is given when required. Those who present with moderate-to-severe dyspnea are given high oxygen concentrations by a Ventimask.

### 2.4. Study Population

The study population included mothers of children who were hospitalized in *Kumudini Women's Medical College *and* Hospital* due to pneumonia. A three-member team of clinicians (1) and research assistants (2) prepared a list of 33 under-5 children who were hospitalized during the last 4 weeks, July-August, 2013; among them, 2 mothers refused to participate. The researcher was assisted by a research assistant. The respondents (mothers) gladly expressed their interest to participate voluntarily in focus group discussion (FGD) sessions and eventually gave their consent in writing.

### 2.5. Sampling Method

Opportunistic and convenient sampling method was used for this study as we required reaching the study participants within the shortest possible time while proportionality was not of primary concern. Thus, we sampled the targeted study population by geographic location so that they could come to Mirzapur township in a group for participating in FGD sessions. The characteristics of these specific predefined group participants were as follows: mothers aged 15 years and more, those whose children have already been identified as recently hospitalized patients through review of hospital logs, and those who were thought to be able to travel and attend in a group at the FGD session.

### 2.6. Instruments/Tools

A guideline was developed based on the concept note as well as literature review.

### 2.7. Pretesting of the Data Collection Tools

The guideline and its necessary modifications were discussed thoroughly during extensive field-testing in rural environments (among a group of mothers of pneumonia children of Mirzapur subdistrict who were hospitalized and residents of non-DSS area).

### 2.8. Methodological Framework

The study was conducted in the month of August 2013. Information was collected on knowledge, perception, and health care seeking behavior of the mothers of pneumonic children. FGD sessions were conducted to reveal information of study interest from a small group of people who came together and shared their ideas, views, and experiences. Mothers were simply asked to share their knowledge and perception about pneumonia and especially of health care seeking behavior. Thus, we obtained insights into the target audiences' perceptions, needs, problems, and beliefs regarding young childhood pneumonia.

Four FGD sessions were conducted, and 7-8 mothers (of enlisted children) were invited to participate in each session. The purpose of FGD was to obtain insights into the contextual issues related to caregivers' perception about pneumonia and its clinical features, causes and healthcare seeking behaviors related to pneumonia, and barriers for seeking timely treatment of childhood pneumonia in rural Bangladesh. FGD sessions were conducted at hospital setting and two separate sessions were arranged on each day. At the beginning, we gave identification number based on random selection to the participating mothers and wrote their name in a piece of paper. They were requested to utter their number each time before expressing their opinions. Each session lasted for 30–45 minutes and discussions were recorded on tape; notes were taken concurrently. Data collection ceased when the point of saturation was achieved and narrative data were rich and had insightful meaning that the additional data would not yield any new information any more.

### 2.9. Data Management and Analysis

Data underwent the process of content analysis. All interviews recorded on tape were transcribed. Themes and subthemes were identified after detailed examination of the narrative texts based on the study objectives. Common themes were identified and key words and phrases from the text were used to mark the themes throughout the text.

Themes were identified under six main sections (i) perception about pneumonia, (ii) knowledge about signs and symptoms of pneumonia, (iii) experience from child's recent illness, (iv) perception about causes of pneumonia, (v) healthcare seeking behavior, and (vi) identification of modifiable barriers in seeking treatment for childhood pneumonia.

### 2.10. Ethical Consideration

The study was approved by both Research Review Committee and Ethical Review Committee of icddr,b. The consent form was written in simple Bengali language (local language) for ease of understanding by the study participants. The consent form was read aloud to the participants if she was unable to read prior to obtaining written consent. Names and identities of the study participants were not used while analyzing data.

## 3. Results

### 3.1. General Information

The median age of the children was 7 months (range from 10 days to 48 months) and 61% (19/31) were male. Mean age of the caregivers was 24.4 ± 5.4 years and 7 of them had no formal education and 3 completed 12 years of schooling. Number of family members ranged between 3 and 12 and 36% (11/31) of the families had at least two children less than 5 years old.

### 3.2. Perception about Pneumonia

 
Table 1 describes the local terminology for clinical features of pneumonia and other related disease. When tried to understand mothers' perception about pneumonia, they opined that pneumonia is a critical disease for young children. It is a severe form of cough and cold (*thanda, kashi*), which develops quickly from cough and cold, and children become weak (*durbol*). If the incidence of cold lasts more than two weeks, then it could result in pneumonia; however it could be prevented. As it is a very bad disease, it can turn into asthma (*hapani*) and the child may also develop heart disease (*harter oshukh*) or child may even die.
   One of the children's mothers clearly spelled out the experience of her child pneumonia and described, “*Pneumonia is a critical disease (khub jotil oshukh), as I know; it is a disease that is associated with cold. If the duration of cold lasts for more than two weeks, then it could result in pneumonia*.”
   Another one also defined pneumonia in her own terminology, “*It is a critical form of cough and cold (thanda kashi). Pneumonia develops quickly from cough and cold (thanda kashi). In the past, it was a serious disease, but nowadays it can be prevented*.”
   A mother defined further pneumonia as follows: “*It is a very bad disease, and it can turn into asthma (hapani), difficulty in breathing (shash nite khub koshto hoy), or shortness of breathing (dom pai na)*.”

### 3.3. Knowledge about the Clinical Features of Pneumonia

- Twenty-eight out of 31 (90%) respondents heard the name of pneumonia before the recent onset of their children's pneumonia; however, three indicated that they had never heard about pneumonia.
- Only nine (29%) respondents had the prior understanding of signs and symptoms of pneumonia. They mentioned that chest retractions (*buk debe jai*), difficulty in breathing (*shash nite koshto hoi*), noisy breathing (*ghor ghor shobdo kore*), and the child stopping taking breast milk or crying a lot (*khub kanna kati kore*) those are the signs and symptoms of pneumonia. They had prior experience with pneumonia because their child suffered from pneumonia before this recent episode. Only one mother (3%) has learnt about these signs and symptoms from both watching television (mass media) and attending clinic (small health facility). Other respondents could not describe the signs and symptoms of pneumonia clearly. They took their children to the hospital because their child had at least two signs and symptoms such as severe cough with running nose, common cold, sneezing, noisy cough, noisy breathing, difficult or rapid breathing, chest retractions, fever with shivering (*kapuni diye jor*), lethargy (*netiye pore*), and high fever with convulsion (*jor er sathe khichuni*).

### 3.4. Recent Experience about Pneumonia

- Mothers of pneumonic children stated that their episode of pneumonia lasted for 7–25 days. The most common symptoms they noticed during their child's pneumonia were cough with running nose, difficulty in breathing or rapid breathing, sneezing, noisy breathing, chest retractions, very cold to touch, bluish or black coloration, skin rash, unable to suck or stopped taking breast milk, and high fever (as high as 102°F or more) with convulsion along with incessant crying. Words used to describe breathing included “fast,” “difficult,” “unusual sound produced by child during breathing,” and “chest retractions”.
   A mother shared her recent experience of her child's pneumonia, “*My child's chest was going down, he had severe coughing (khub kashi), sometimes used to vomit during coughing and had difficulty in breathing (shash nite koshto hoi), and during breathing, he opened his mouth and sometimes he took long breath.*”
- The first signs and symptoms that appeared at the beginning of pneumonia were cough, vomiting during coughing, sneezing, running nose, high fever, excessive sweating, incessant crying, unable to suck breast milk, irritable or restless, difficulty in breathing, skin rashes, and convulsion.

### 3.5. Perception about Causes of Pneumonia

- Mothers had the perception that environmental factors such as dust, unhealthy household condition, and cold allergy, heavy sweating during hot summer months, mother with cough in the family, too cold winter months, pouring lots of water on child's head for high fever or sponging whole body with cold water, drinking cold water, and frequent or longer playing with water were the causes for pneumonia among the children.
   A mother gave clear account of her perception about the causes of pneumonia, “*Environmental factors such as dust particles, unhealthy household condition, drinking cold water (including water from freeze) by the child, high temperature during hot summer months causes the child to sweat a lot, and drying of those sweats may cause my child to develop cough*.”
   Another mother also added some causes of pneumonia, “*Allergy, sleeping below ceiling fan, cold and high temperature, and inhalation of dust are responsible for pneumonia*.”
   An additional pneumonia child's mother perceived, “*I also work with cold water (thanda pani), as I breastfeed my child, so he develops cough. Elderly people often speak up that if mother has cough, child also develops cough through breast milk during sucking*.”

### 3.6. Healthcare Seeking Behaviors

- Table 2 illustrates common treatment methods and place of treatment for pneumonia reported by the respondents. While identifying concerns mothers had about child's illness, caregivers stated that they knew instinctively that their child was unwell. Caregivers explained that they know when their child is well and when their child is sick and particularly when something is wrong. The unique knowledge they had about clinical features of severity of disease influenced children's parents or caregivers to decide to take their child to the hospital.
- Healthcare seeking behavior depends on various factors and a mother exclaimed, “*My child had high fever with the symptom of convulsion (jor-er-sathe khichuni). I am a mother, so I can realize the problem of my child and to save my child's life, I decided to take him to hospital.*”
- A very conscious mother explained her child's illness and care seeking behavior, “*My child had problems like rapid breathing (ghono ghono shash tana). I counted that and he had 59 breathes per minute, and then I decided to take him to the hospital.*”
- Further, one mother explained healthcare seeking behavior of her child pneumonia which indicates traditional healthcare behavior of rural Bangladesh, “*My child had cough for 17 days. First, I bought medicine from a local drug store, but he did not recover, then I took him to a small clinic, they gave medicine, but it also did not work. He developed cough with unusual sound inside his chest (ghor ghor shobdo hoi), so I took him to the hospital.”*
- After recognizing abnormal behavior and physical symptoms, caregivers quickly consulted a health care provider or doctor, although all the children received treatment within 24 hours to 20 days from *Kumudini Women's Medical College *and* Hospital*. This is true for both the children who were referred by community level health care providers or self-referred by primary caregivers/mothers to the hospital. Active decision makers or individuals influencing the mothers' decisions were the mothers' themselves, father, community health workers, grandparents, and other family members. They advised mothers to take the child to hospital. Only one mother was advised by qualified doctor to take her child to the hospital.
- Only nine mothers (29%) could properly diagnose the signs and symptoms of pneumonia, because they had previous experience with childhood pneumonia. Families with history of death of any child due to pneumonia and similar attacks of pneumonia were reported by other children of the family or children living in the immediate neighborhood. Repeated pneumonia in same child is not uncommon. Other mothers could not diagnose whether their child had pneumonia or not. Respondents visited local drug store (for allopathic or homeopathic medicine) or clinic or hospital. Thirteen caregivers (42%) came straight to the hospital whereas other eighteen (58%) respondents tried to treat their child at home by procuring medicine from local drug store. Traditional health remedies were given initially by two caregivers for pneumonic illnesses as they felt that those would be appropriate. However, mothers of ten children (33%) gave both herbal preparation as well as allopathic or homeopathic medicine obtained from local small drug store.
- Different types of traditional treatment methods were started at household levels initially for treatment of pneumonia such as spiritual inhaling (*jhara deya*), herbal syrup, massage of any herbal medicine called “telachuna” on palm and sole of the child, and also massage of kerosene oil on the chest of the child. They usually applied or gave those treatments 2–4 times in a day to the child. They narrated that elderly people and community health workers advised them to give or apply those herbal medicines to their child, and they indicated that they thought those would be good for their children's cough. The following quotes illustrate the treatment methods that were applied during childhood pneumonia:
   Traditional and initial treatment method was illustrated by one pneumonia child's mother that she applied to her child during pneumonia, “*Elderly people told that tulshi leaf (herbal medicine) with breast milk is good for cough and cold, so first I gave tulshi leaf with breast milk to my child; but once I observed that his condition is getting worse, and I stopped giving breast milk. However, my baby suddenly developed convulsion, and I took him to the hospital immediately.”*
   Another mother also shared, “*My child had symptoms like running nose, cough, and cold. Initially, I gave him herbal medicine (lemon juice and extracts of tulshi leaf with lukewarm water) 3-4 times per day and I also bought medicine from local drug store, but my child did not get well. Then I took him to the hospital.*”
   One mother applied multiple traditional treatment methods to her child during pneumonia and she expresses, “*I gave the same herbal medicine (lemon juice and extracts of tulshi leaf with lukewarm water) mixing with misri (condensed sugar) twice a day to my child. I also massaged herbal medicine called “telachuna” (calcium carbonate (chun) and mustard oil heated on mango leaf) on palm and sole of my child twice a day. It kept my child warm. My mother-in-law and other elderly individuals of the family advised me about those remedies.*”

### 3.7. Modifiable Barriers to Pneumonia Treatment

 Those caregivers, who delayed in reporting to the hospital, were asked about the reasons or the barriers that caused delay in seeking health care from the facility. The explanations for barriers that stopped parents to take their child to a facility on time were as follows: they perceived that the illness was not serious enough requiring reporting to health facility or lack of money for treatment of their child or long distance has to be travelled or poor road condition causing travelling to be difficult or time consuming or waiting time in hospital. Moreover, mothers stated that they alone could not bring their children to the hospital and they waited for another person for accompanying her. Two of the respondents opined that their in-laws (father-in-law, mother-in-law, or sister-in-law) did not like that they take their child to the hospital, to them (in-laws) it was unnecessary. Five caregivers (16%) shared their views that when they were able to borrow some money from others, they came to hospital for their child's treatment.

## 4. Discussion

In developing countries including Bangladesh, mothers or primary caretakers of pneumonic children had inadequate knowledge about pneumonia [2, 11–13]. Most of them could not recognize whether their child had pneumonia or not. When the mothers detected that their child either was not breathing properly or had high fever or convulsion or was unable to take or stopped taking or was reluctant to receive the feed, they brought their child to the health facility. Majority of them did not have prior knowledge about the clinical features of pneumonia. Those mothers who had knowledge about clinical features especially early danger signs of pneumonia, they even could not identify properly that their children were suffering from pneumonia. They brought their child to the hospital when the child developed late danger signs with severe form of the disease or associated complications. It is very important that rural mothers should have appropriate knowledge about the clinical features of pneumonia, because delays in detecting clinical signs including danger are the major obstacles to preventing deaths due to childhood pneumonia. It has been observed that mothers were unable to detect the severity of the illness of their child and brought the matter to the attention of adult family members or household head in order to get permission to take the child outside of home for treatment. A study in recent past in western Kenya reported that comorbidities, spread from upper respiratory tract and delay in seeking treatment, were the common identified causes of severe pneumonia on presentation to health facilities [7].

In the present study, the median age of pneumonic children was 7 months (range of 10 days–48 months), which implies that under-five children of any age range including newborns are at risk for pneumonia. The younger the child, the higher the risk of death from pneumonia [14]; therefore, institution of appropriate treatment should not be delayed, as one mother opined that her elder child died due to late pickup of clinical features of pneumonia. Another observation made was that those children who had pneumonia in the recent past, they experienced attacks of pneumonia again. An unpublished data revealed that previous history of pneumonia is one of the major risks for pneumonia related deaths in the community (Mohammod Jobayer Chisti—unpublished, personal communication). So, appropriate health education including recognition of the danger signs and symptoms, institutional supports, and government initiatives at community level may reduce the incidence of pneumonia.

Environmental factors such as dust, unhealthy household condition, and high room temperature during hot summer months, cold allergy, and winter seasons were perceived as the causes of pneumonia in the present study. In rural Bangladesh, child usually comes in contact with smoke especially produced while cooking food, which is a known risk factor for pneumonia [15–17]. Moreover, a group of mothers believed that if they have cough and cold, their child would also develop cough through breastfeeding while sucking breast milk. There is no adequate data on spread of pneumonia from the infectious cough of breastfed mothers, although breastfed mothers with active tuberculosis are one of the risk factors for developing tuberculosis in children [18]. However, proper breastfeeding can prevent the severity of pneumonia such as hypoxemia [18]. This finding implies that mothers have lack of appropriate knowledge about pneumonia and its prevention. Childhood pneumonia is associated with poverty and results from suboptimal child rearing and care seeking practices compounded by lack of access to healthcare [19]. Previous studies reported that hand hygiene practices are vital in minimizing the spread of most organisms responsible for pneumonia, which can reduce the incidence of acute respiratory infections and pneumonia by up to 50 percent [19–21]. Air pollution, maternal illiteracy, and unfamiliarity with respiratory illnesses are the risk factors for childhood pneumonia [19, 22] which are supportive of present study findings about the knowledge gap and lack of appropriate perception of mothers about pneumonia.

Home medication was relatively common in the present study; however, appropriate home management of ARI prevents serious consequences of pneumonia, but inappropriate home medication is dangerous [9, 23]. Children who received traditional treatment delayed to report to the hospital including parents who sought treatment from drug stores or local unlicensed health care providers [24]. Reasons behind delayed reporting were: illness was not perceived as serious enough requiring reporting to the health facility by the mothers or lack of money for treatment of their children, long distance or deleterious road condition or long waiting times in the facility. There were some social or household level barriers that stopped mothers from taking their child to health facilities on time. Sometimes elderly people discourage parents from seeking appropriate treatment for their children, which is really a serious concern for appropriate and timely treatment of childhood pneumonia. To the elderly people, herbal or spiritual inhalation is the appropriate treatment for pneumonia or cough and cold. Although, different types of traditional treatment methods are applied initially at household level for pneumonia treatment by mother herself or as and when advised by elderly family members and/or community health care providers. According to mothers, those treatments were only appropriate when their child had cough and cold. Previous studies accounted that vitamin C has an effective role in pneumonia treatment in those who have less serum vitamin C, as it influences immune system [25] and the metabolism of vitamin C is changed in infectious diseases including pneumonia, as indicated by decreased levels in plasma, leucocytes, and urine [25–27]. In the present study mothers used lemon juice and tulsi leaf (green leaf) as both contain vitamin C. So, those herbal remedies might have some effective role in pneumonia, although we did not measure the effects. According to integrated management of childhood illness (IMCI) lessons, the caregivers should be taught to monitor any of the danger signs of pneumonia during their attempts to treat their kids keeping at home. In developing countries like Bangladesh, most of the people are deprived from access to basic health care which is a fundamental right [28]. Accessibility to medical care is often interrupted because of location, financial requirements, lack of technical support to patient population, social distance between client and provider, and preference for male child; this may explain the unusual gender ratio of 61% [28]. So, initiatives to strengthen service delivery at existing small rural health facilities for treatment of childhood pneumonic children with appropriate referral system are important. Health education, particularly orientation of community members on danger signs and symptoms of childhood pneumonia, of utmost importance. Despite its strengths, this study has several limitations. First, this study employed a convenience sample of women who sought care from a rural facility of Bangladesh for their child's recent pneumonia. The study was designed to understand qualitative perspectives of a rural community, thus qualitative results cannot be generalized widely. Additionally women sought treatment for their pneumonic children in this hospital, thus, questions regarding the inclusion of mothers into the study for FGD who did not visit the same facility with their pneumonic child seemed difficult to answer. During FGD sessions, attempts were made to ensure active participation of each and every member in sharing ideas, views, and experiences. Another limitation of the study is that we have no data on infants with pneumonia who died before arriving to the hospital.

## 5. Conclusions

Most of the rural mothers did not have adequate or appropriate knowledge or perception about childhood pneumonia and did not seek proper care even when their child was suffering from severe pneumonia or very severe pneumonia or very severe disease. Health education programs at household or community level or mass education campaigns should be implemented to disseminate knowledge about the signs and symptoms including danger signs of pneumonia requiring immediate treatment at facility level and preventive measures against childhood pneumonia.

## Figures and Tables

**Table 1:** Local terminology for clinical features of pneumonia and other related diseases.

Terminology:	
Clinical features:	
Fever-*jor *	
Cough-*kashi *	
Sneezing-*hachi *	
Noisy cough-*dom dom shobdo kore *	
Noisy breathing-*ghor ghor shobdo kore *	
Running nose-*nak diye pani pore *	
Red skin rashes-*lal lal gota *	
Difficult breathing-*shash tante or shash nite koshto hoi or* *nishash tante koshto *	
Rapid breathing-*ghono ghono shash tane *	
Chest retractions-*buk debe jai, buk venge jai *	
Convulsion-*khichuni* Fever with shivering-*kapuni diye jor* Lethargic-*Netiye pore *	
Diseases:	
Common cold-*thanda* Asthma-*hapani *	
Heart disease-*harter oshukh *	
Other Terms:	
Cold (disease, food/drink, temperature)	
Cold to touch-(*hat paa thanda*)	
Changing of body color (bluish or black)-*gayer rong nil,* *kalo hoye jay *	

**Table 2:** Common treatment methods and place of treatment for pneumonia reported by the respondents.

Treatment methods	Application	Preparation and Bengali term	Place of treatment
(1) Spiritual	Spiritual inhaling	Jhara deya	Community inhaler

(2) Traditional	Herbal syrup or remedies	Lemon juice and extracts of tulsi leaf with lukewarm water with or without condensed sugar (*misri*)	At home
	Massage of herbal medicine	(i) Telachuna (calcium carbonate (*chun*) and mustard oil heated on mango leaf) (ii) Kerosene oil	At home

(3) Homeopathic	Globules		Local drug store

(4) Allopathic	Antimicrobial suspension or injectable, bronchodilator, cough depressant, and antihistamine suspension		(i) Local drug store (ii) Private clinic (iii) Hospital
